# New Class of Monoclonal Antibodies against Severe Influenza: Prophylactic and Therapeutic Efficacy in Ferrets

**DOI:** 10.1371/journal.pone.0009106

**Published:** 2010-02-08

**Authors:** Robert H. E. Friesen, Wouter Koudstaal, Martin H. Koldijk, Gerrit Jan Weverling, Just P. J. Brakenhoff, Peter J. Lenting, Koert J. Stittelaar, Albert D. M. E. Osterhaus, Ronald Kompier, Jaap Goudsmit

**Affiliations:** 1 Crucell Holland BV, Leiden, The Netherlands; 2 ViroClinics BV, Rotterdam, The Netherlands; 3 Department of Virology, Erasmus MC, Rotterdam, The Netherlands; Tsinghua University, China

## Abstract

**Background:**

The urgent medical need for innovative approaches to control influenza is emphasized by the widespread resistance of circulating subtype H1N1 viruses to the leading antiviral drug oseltamivir, the pandemic threat posed by the occurrences of human infections with highly pathogenic avian H5N1 viruses, and indeed the evolving swine-origin H1N1 influenza pandemic. A recently discovered class of human monoclonal antibodies with the ability to neutralize a broad spectrum of influenza viruses (including H1, H2, H5, H6 and H9 subtypes) has the potential to prevent and treat influenza in humans. Here we report the latest efficacy data for a representative antibody of this novel class.

**Methodology/Principal Findings:**

We evaluated the prophylactic and therapeutic efficacy of the human monoclonal antibody CR6261 against lethal challenge with the highly pathogenic avian H5N1 virus in ferrets, the optimal model of human influenza infection. Survival rates, clinically relevant disease signs such as changes in body weight and temperature, virus replication in lungs and upper respiratory tract, as well as macro- and microscopic pathology were investigated. Prophylactic administration of 30 and 10 mg/kg CR6261 prior to viral challenge completely prevented mortality, weight loss and reduced the amount of infectious virus in the lungs by more than 99.9%, abolished shedding of virus in pharyngeal secretions and largely prevented H5N1-induced lung pathology. When administered therapeutically 1 day after challenge, 30 mg/kg CR6261 prevented death in all animals and blunted disease, as evidenced by decreased weight loss and temperature rise, reduced lung viral loads and shedding, and less lung damage.

**Conclusions/Significance:**

These data demonstrate the prophylactic and therapeutic efficacy of this new class of human monoclonal antibodies in a highly stringent and clinically relevant animal model of influenza and justify clinical development of this approach as intervention for both seasonal and pandemic influenza.

## Introduction

A novel class of human monoclonal antibodies against influenza has been recently discovered [1]. These antibodies bind to the membrane-proximal stem of haemagglutinin, the major viral surface protein, and neutralize the influenza virus by blocking its fusion with the host cell [2]. A panel of antibodies with a similar mode of action was reported subsequently by Sui *et al*. [3]. Due to the high conservation of their recognition site, this class of antibodies has shown the ability to neutralize a broad spectrum of influenza subtypes, including H1, H2, H5, H6 and H9 [1], and can be expected to also neutralize viruses from subtypes H4, H8, H11–H14 and H16, as well as their future antigenic drift variants [2]. One of these antibodies, CR6261, was investigated in mice and shown to be protective when given before and after lethal challenges with H1N1 and H5N1 virus, suggesting that it has potential as the first-ever broad-spectrum monoclonal antibody for prophylaxis and treatment of influenza virus infections [1].

According to the World Health Organization, seasonal influenza causes up to 500,000 deaths worldwide each year [4]. Immunologically naïve infants, immunocompromised individuals and the elderly are particularly susceptible to illness caused by seasonal influenza viruses, with 90% of deaths occurring in the latter group [4], [5]. In addition, subtypes of influenza A viruses that have not previously circulated among humans occasionally cross from animal reservoirs, raising the spectre of a pandemic.

Over the past decade, the most prominent pandemic threat appeared to be posed by highly pathogenic avian influenza viruses of subtype H5N1. However, it was a new strain of human H1N1 that emerged in Mexico and the United States in March and April 2009 and rapidly spread across the globe that caused the WHO to declare a pandemic on June 11th [6], [7]. Although the virulence of this virus is currently moderate, particularly compared to the case fatality rate of over 60% of human H5N1 infections, this may change over time. Meanwhile, the threat from highly pathogenic avian H5N1 viruses persists as they continue to circulate and evolve in bird populations.

Preventive vaccination has historically been the primary means of influenza control, but this approach has important limitations. Vaccines typically elicit a potent neutralizing antibody response only to the specific viral strains they contain, and closely related viruses [8], [9]. Furthermore, influenza vaccines have suboptimal immunogenicity and efficacy in the groups at highest risk of severe disease: the very young, the elderly and immunocompromised individuals [10].

The current therapeutic regimen for influenza A is limited to two classes of drugs: the adamantanes (amantadine and rimantadine) and the neuraminidase inhibitors (oseltamivir and zanamivir). Adamantanes rapidly elicit viral resistance, and resistance rates are high among H3N2 viruses and certain clades of H5N1 viruses [11]–[14]. The use of oseltamivir, the leading antiviral influenza drug, has been limited by the sudden and widespread emergence of resistance among circulating H1N1 influenza strains [15], [16]. Oseltamivir resistance has also been observed during treatment of H5N1 infection [17], [18]. Zanamivir is still effective against H1N1 viruses and resistance to this drug is less likely to arise [19]. However, its use is limited to patients who can actively use an inhaled drug, which excludes young children, impaired older adults, or patients with underlying airway disease [16]–once again, the group of patients most vulnerable to serious complications from influenza infection.

In the absence of reliable antiviral drugs and vaccines, development of alternative strategies for influenza prophylaxis and therapy is urgently required. In order to assess whether CR6261 may be a viable option for human treatment, we evaluated the prophylactic and therapeutic efficacy of this human monoclonal antibody in a highly stringent lethal H5N1 influenza ferret model. The ferret is the most suitable disease model for human influenza infection as it displays very human-like disease [20]–[22].

## Results

### Prophylactic Efficacy of CR6261 against H5N1 Challenge

Four groups of 6 ferrets each received 30, 10, 3 or 1 mg/kg CR6261 via an intravenous injection and were challenged the next day with the highly pathogenic avian A/Indonesia/5/2005 (H5N1) virus. A control group of 6 ferrets received 30 mg/kg of the irrelevant isotype-matched antibody CR3014.

**Figure 1:**
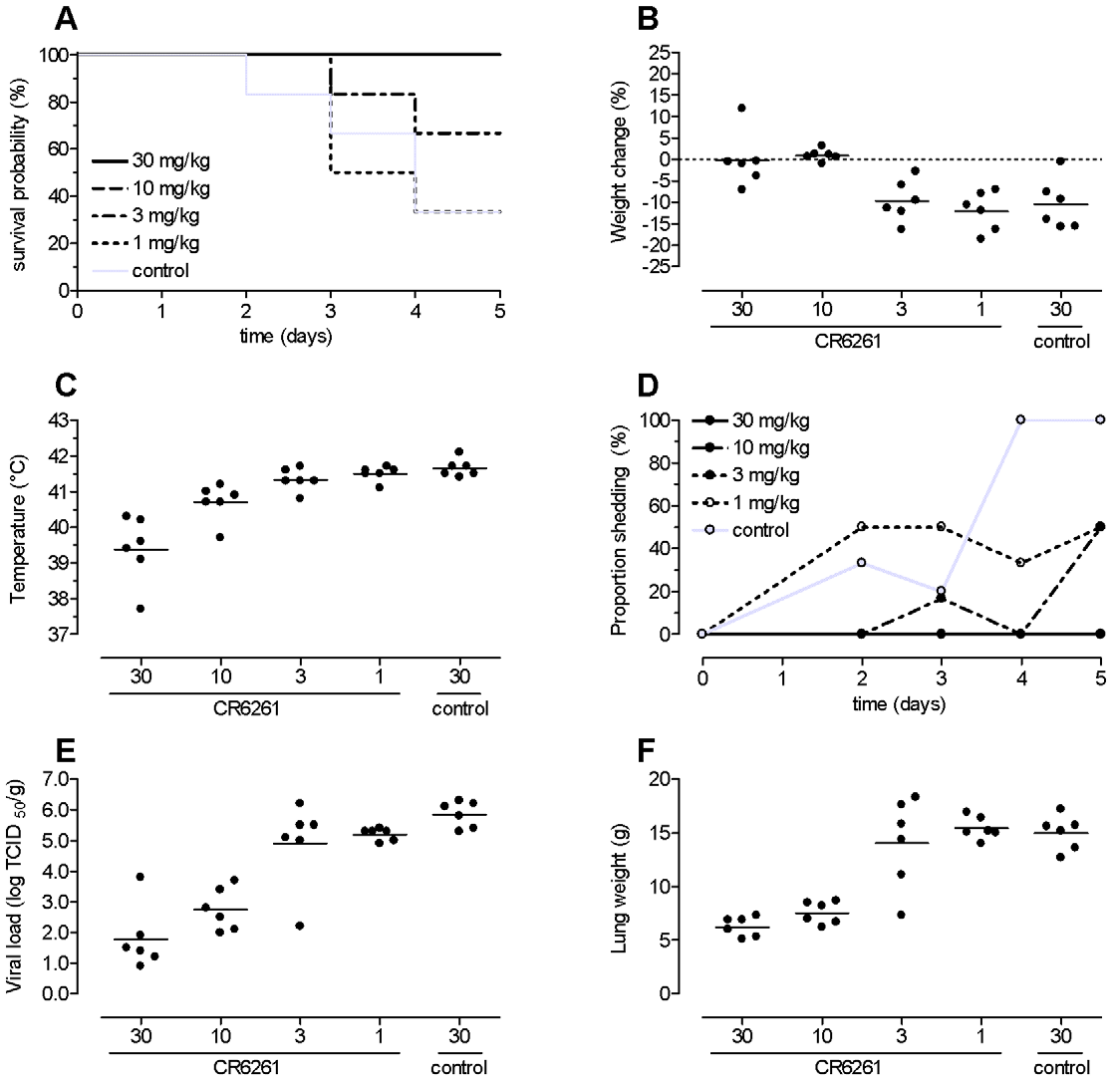
Prophylactic efficacy of CR6261 against lethal H5N1 challenge. Groups of 6 ferrets received 30, 10, 3, or 1 mg/kg of mAb CR6261 or 30 mg/kg control mAb by intravenous injection and were challenged 24 hours later with 10^5^ TCID_50_ of influenza A/Indonesia/5/2005 (H5N1). Ferrets were monitored for 5 days or until death. Panel A: Kaplan–Meier survival probability curves. Panel B: Change in body weight at the end of study (or at death if the event occurred earlier) expressed as percentage from baseline body weight. Panel C: Maximal body temperature observed during the day after challenge. Panel D: Viral shedding of infectious virus in the upper respiratory tract. The graph shows the proportion of ferrets alive with infectious virus detected in nasal and/or throat swabs. Panel E: Viral load in lung tissue as determined by virus titration on MDCK cells. Panel F: lung weights as determined after necropsy. Dots in panels B, C, E and F represent individual animals; group means are indicated by the horizontal lines.

All ferrets that received 30 or 10 mg/kg CR6261 survived, compared to only 33.3% of the control animals (Figure 1A). A further reduction of the dose to 3 mg/kg was clearly correlated with a lower survival rate of 66.7%. Though not statistically significant different from survival observed in control animals, there is a 50% mortality reduction. The lowest dose of 1 mg/kg CR6261 was not associated with survival benefit compared to control animals. Survival times differed significantly between groups receiving 30 or 10 mg/kg CR6261 and the control group (p = 0.020).

Moribund animals showed general depression, anorexia and lethargy, and exhibited clinical signs of respiratory disease, including dyspnoea. Animals treated at efficacious dose levels (30 and 10 mg/kg) did not loose body weight, whereas the mean weight loss in the control group was 10.5% by the time the ferrets died or were euthanized (Figure 1B). An exception was one control ferret that succumbed to infection within 48 hours after challenge and lost hardly any weight before. Ferrets that received 3 or 1 mg/kg CR6261 showed similar declines in body weight as the control animals.

One day after challenge the maximum body temperature was observed; for each ferret the maximum body temperature is depicted in figure 1C. The groups treated with 30 or 10 mg/kg had mean temperatures of 39.4°C and 40.7°C, respectively, which were significantly lower than the mean of 41.7°C observed in the control group (p<0.001 and p = 0.015, respectively). The mean temperature observed in animals treated with the lower doses of CR6261 (41.3°C and 41.5°C for 3 and 1 mg/kg, respectively) did not differ significantly from the control group.

The mean temperature in animals observed 3 days before challenge was similar across the 5 groups, ranging from 37.6°C to 38.4°C. Individual body temperature varied considerably within one healthy ferret over 24 hours (standard deviation 0.7°C).

Ferrets treated with 30 or 10 mg/kg CR6261 did not shed infectious virus in the upper respiratory tract at any time, whereas animals in the control group did (Figure 1D). Treatment with 3 and 1 mg/kg did not prevent shedding, but reduced the proportion of ferrets with infectious virus in nasal and/or throat swabs.

After necropsy, all ferrets were assessed for viral load in the lungs (figure 1E), and the animals that received 30 mg/kg CR6261 or control antibody were also assessed for viral load in the brain, liver, spleen, blood and kidney. The levels of virus replication in the lungs of ferrets treated with 30 and 10 mg/kg CR6261 were 3.9 and 2.9 log_10_ TCID_50_/g lower than that in the control group (both p<0.001). No infectious virus was detected in any of the other organs of the ferrets that received 30 mg/kg CR6261, whereas infectious virus was found in the brain of 2, the liver of 3, and the spleen of 5 of the 6 control animals (data not shown). There was no significant difference in lung viral load in the groups receiving 3 or 1 mg/kg CR6261 compared to the control group (p = 0.74 and p = 0.91, respectively).

Histopathological results were in accordance with the findings described above; animals that received 30 and 10 mg/kg CR6261 showed much less pulmonary lesions such as primary atypical pneumonia, subacute bronch(iol)itis, emphysema and congestion, or showed such lesions at a lower grade of severity, compared to animals from the other groups. Bronchiolitis obliterans was not observed in any animal that received 30 mg/kg CR6261. Compared to this higher-dose group, animals that received 10 mg/kg CR6261 showed more regenerative response (diffuse grey/red area and bronchioloalveolar hyperplasia) in the lungs, and more inflammatory changes in the trachea. Pulmonary oedema was not observed in animals that received 30 or 10 mg/kg CR6261, but was observed frequently in all other groups. These findings were in agreement with mean lung weights, which were lowest in the animals treated with 30 or 10 mg/kg CR6261 (6.3 g and 7.6 g, respectively) and significantly lower in this group than in control animals (15.0 g; both comparisons p<0.001). No significant difference in lung weight was found between the groups receiving 3 or 1 mg/kg CR6261 (14.1 g and 15.4 g, respectively) and the control group (15.0 g).

These findings show that, in a dose dependent way, prophylactically administered CR6261 confers protection against lethal H5N1 challenge, prevents morbidity and viral dissemination and reduces pulmonary pathology.

### Therapeutic Efficacy of CR6261 against H5N1 Challenge

To assess the therapeutic efficacy of the monoclonal antibody CR6261, two groups of 10 ferrets were challenged as above and given 30 mg/kg of CR6261 either 4 or 24 hours later. A comparator group of 10 ferrets received 30 mg/kg of the control antibody 4 hours after challenge.

**Figure 2:**
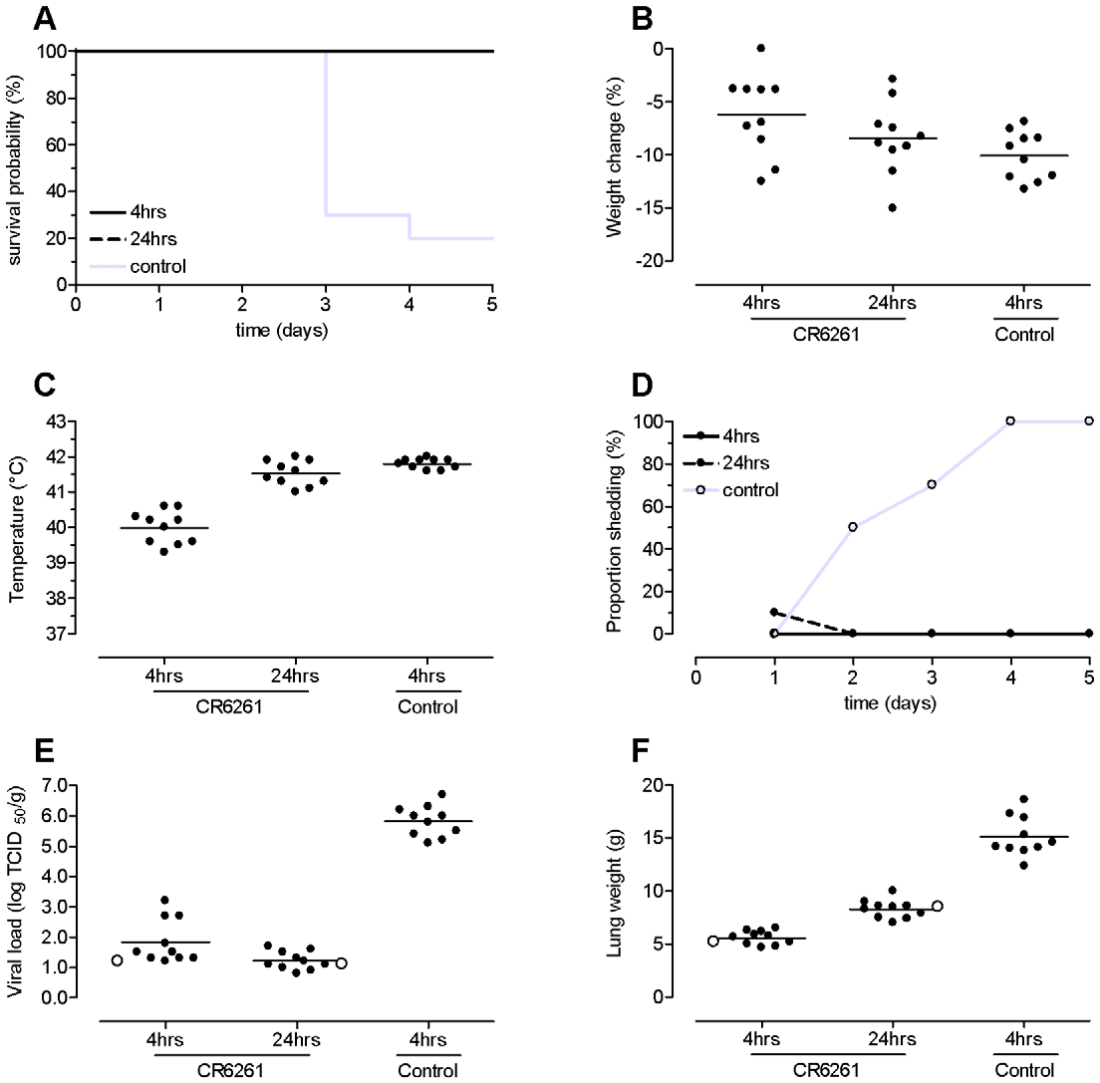
Therapeutic efficacy of CR6261 against lethal H5N1 challenge. Groups of 10 ferrets received 30 mg/kg mAb CR6261 by intravenous injection either 4 or 24 hours after challenge with 10^5^ TCID_50_ of influenza A/Indonesia/5/2005 (H5N1) virus. A control group of 10 ferrets received 30 mg/kg of a control mAb 4 hours after challenge. Ferrets were monitored for 5 days or until death. Panel A: Kaplan–Meier survival probability curves. Panel B: Change in body weight at the end of study (or at death if earlier) expressed as percentage from baseline weight. Panel C: Maximal body temperature observed the day after challenge. Panel D: Viral shedding of infectious virus in the upper respiratory tract. The graph shows the proportion of ferrets alive with infectious virus detected in nasal and/or throat swabs. Panel E: Viral load in lung tissue as determined by virus titration on MDCK cells. Panel F: lung weights as determined after necropsy. Dots in panels B, C, E and F represent individual animals; group means are indicated by horizontal lines. The open circles (panel E and F) indicate one additional ferret per CR6261 group that was euthanized at day 3 for virus isolation and pathology.

Survival rates in the groups receiving CR6261 at 4 and 24 hours after challenge were 100%, whereas only 20% of the animals in the control group survived (p<0.001) (Figure 2A). Mean decline in body weight at the end of the experiment was 6.2% in the group of ferrets that received CR6261 4 hours after challenge (Figure 2B), which was significantly less (p = 0.025) than the 10.1% observed in control animals. Animals treated 24 hours post challenge showed a mean body weight loss of 8.4%, which was not significantly different from the control animals (p = 0.427). The group of ferrets treated with CR6261 4 hours post challenge had a mean maximum temperature of 40.0°C, compared to 41.8°C in the control group (p<0.001). In line with the rapid rise in temperature after challenge observed in the prophylaxis experiment ferrets treated with CR6261 24 hours after challenge showed a mean maximal temperature of 41.5°C before CR6261 was administered (p = 0.15 versus 41.8°C of the control group, figure 2C).

Ferrets treated with CR6261 at 4 hours post challenge did not shed infectious virus in the upper respiratory tract throughout the study (figure 2D). In the group treated with CR6261 at 24 hours post challenge, one ferret had a low concentration of infectious virus (2.8 log_10_ TCID_50_) in the throat on day one, but no virus was detected on subsequent days. In contrast, all animals in the control group shed virus during one or more days. Accordingly, the mean viral loads in the lungs of ferrets treated with CR6261 at 4 and 24 hours post challenge were considerably lower than that in the control group (differences were 3.9 and 4.5 log_10_ TCID_50_/g, respectively, both comparisons p<0.001; Figure 2E).

The lungs of animals that received CR6261 at 4 hours post challenge showed less pulmonary lesions (alveolar oedema, bronchiolitis obliterans, congestion, emphysema, bronchioloalveolar hyperplasia and primary atypical pneumonia), or showed such lesions at a lower grade of severity, compared to the lungs of animals from the other two groups. Animals of the control group were most affected by primary atypical pneumonia. These findings were in accordance with the observation that the mean lung weights of ferrets treated with CR6261 at 4 hours post challenge were lower compared to the control group (5.7 g versus 14.9 g, p<0.001; Figure 2F). Animals that received CR6261 at 24 hours post challenge showed most regenerative response (bronchioloalveolar hyperplasia) in the lungs, suggesting damage to the lung parenchyma with subsequent regenerative response. The mean lung weight in this group was significantly higher than that of the group receiving CR6261 at 4 hours post challenge (8.4 g versus 5.7 g), but lower than that of the control group (p<0.001).

From the study outset, one animal had been added to each of the two treatment groups to be sacrificed for gross-pathology and histology on lungs as soon as 50% of the control animals died. The purpose was to test for possible bias due to differences in the timing of death. Infectious virus titres in the lungs of the treated ferrets sacrificed at day 3 were identical to those in treated animals sacrificed at the end of the study (open circles in figure 2E). Similarly, there were no differences in lung weight and pathology between animals sacrificed at day 3 or after day 5 (figure 2F). This indicates that the results were not biased by differences in the timing of euthanasia or spontaneous death.

## Discussion

CR6261 represents a new class of human monoclonal antibodies that exhibits immediate and potent efficacy for the prevention or treatment of influenza in a clinically relevant model for severe disease. The results presented here also confirm previously reported data demonstrating the prophylactic and therapeutic efficacy of CR6261 in mice challenged with H5N1 influenza viruses. Together with the H1N1 challenged mice data published earlier [1] these findings indicate that CR6261 is effective across a broad spectrum of influenza viruses, including seasonal and potentially pandemic strains.

Passive immunotherapy would be particularly beneficial for the groups at highest risk of severe disease due to seasonal influenza–the elderly and immuno-compromised–but may also be indispensable for the general public in the event of a pandemic disease outbreak caused by high-risk pandemic candidates such as H2, H5, H6 and H9.

Monoclonal antibodies against influenza viruses have been studied for decades, but their potential–and thus development–as ‘passive’ immunotherapy for influenza has been inhibited by the lack of monoclonal antibodies with broad neutralizing activity. This lack is due to the tolerance of influenza virus for genetic changes in the most immunogenic regions on its surface. The recent discovery of broadly neutralizing human monoclonal influenza antibodies [1] and the demonstrated efficacy of a representative of this novel class of antibodies against lethal viral challenge in a clinically relevant model, as presented in this paper, create an opportunity for the prevention and treatment of influenza infections, regardless of the causal strain. The possibility of a potent and broadly neutralizing agent that would equip clinicians and public health workers to deal effectively with the influenza viruses of the future represents a paradigm shift in the approach to influenza control.

Influenza illness observed in the ferrets infected with H5N1 virus in this study closely resembles influenza in humans with H5N1 infection, who present with fever, cough, shortness of breath, and radiological evidence of pneumonia [23]. Besides respiratory symptoms, gastrointestinal symptoms such as diarrhoea, vomiting and abdominal pain are often present. In severe cases, the pneumonia rapidly progresses to acute respiratory distress syndrome and multiorgan failure. High viral loads, extrapulmonary virus dissemination and hypercytokinaemia are associated with fatal outcome (reviewed in [24]–[26]). Autopsies of patients who succumbed to influenza A (H5N1) virus infection have shown diffuse alveolar damage, patchy interstitial lymphoplasmacytic infiltrates, bronchiolitis with squamous metaplasia and pulmonary congestion with various degrees of haemorrhage [27]–[29]. The clinical signs observed in the control animals of this ferret study correspond to the most severe influenza pathology in humans [23]. The efficacy of CR6261 in preventing these clinical signs in the prophylactic and therapeutic ferret model presented here, together with the efficacy shown in mice after lethal challenges with different viruses, strongly indicate that CR6261 can be expected to be efficacious against disease caused by the other, less virulent viruses it neutralized in vitro [1].

In a meta-analysis of experimental influenza infection of placebo-treated and untreated healthy volunteers, Carrat *et al.* [30] studied 1280 participants who were challenged with either influenza type H1N1, H3N2 or B. Interestingly, viral shedding was highly correlated with the presence of clinical symptoms such as fever, runny nose, sore throat, sneezing, cough and shortness of breath. The authors concluded from their meta-analysis that subjects with symptomatic illness shed virus in amounts 100 to 1000 fold higher than subjects who were not ill. The fact that CR6261 after intravenous administration instantaneously reduces viral shedding indicates that in man CR6261 might reduce clinical symptoms in subjects infected with influenza virus. In addition, studies in guinea pigs showed that a reduction in nasal wash titers correlate with a decreased efficiency of viral transmission by aerosol [31]. The ability of CR6261 to abolish shedding of virus in pharyngeal secretions strongly suggests that antibodies like CR6261 might prevent or reduce virus spreading at the onset of an epidemic influenza outbreak in nursing homes or in case of a pandemic.

In this study, we assessed the efficacy of the human monoclonal antibody CR6261 against a highly pathogenic avian H5N1 virus, as this provides a stringent disease model for severe influenza. However, the potential use of this antibody is not limited to viruses of this subtype or to other avian strains that may pose a pandemic threat. CR6261 has been shown to recognize H1 viruses that have emerged over a time span of 90 years from the H1N1 virus which caused the 1918 ‘Spanish flu’ pandemic to the latest Brisbane viruses. Since the epitope is conserved CR6261 is predicted to bind to future antigenic drift variants. This means that CR6261 could be used to protect against all H1 influenza viruses, including the ones resistant to oseltamivir. Use of CR6261 in combination with effective medication against H3, such as oseltamivir or zanamivir, would effectively protect against all seasonal influenza viruses, without a need for prior knowledge of the virus subtype or strain.

In the present study ferrets were challenged with an inoculum which is probably much higher compared to the viral exposure in naturally infection in humans. The rapid deterioration in ferrets with death occurring within 3 days underpins this hypothesis since humans exposed to H5N1 develop the first symptoms 2–4 days after the last exposure and even periods of up to 8 days have been reported [25]. The clinical signs in this model are quite extreme, but important for establishing the efficacy of the antibody as proof of concept. The data of these two experiments demonstrate the prophylactic and therapeutic efficacy of this new class of human monoclonal antibodies in a highly stringent and clinically relevant ferret model of human influenza.

## Materials and Methods

### Antibody

The human monoclonal antibody CR6261 was isolated from the IgM+, CD27+ B cell repertoire of a healthy individual who was recently vaccinated with the seasonal influenza vaccine, using phage display selection on recombinant H5 haemagglutinin [1]. CR3014, an isotype-matched antibody with the ability to neutralize SARS corona virus–which has similar tissue/organ tropism as that of H5 viruses–was used as a control antibody [32]. Both antibodies were produced on PER.C6® cells.

### Animal Studies

The study was performed with outbred ferrets (*Mustela putorius furo*, female, age approximately 8 months, Schimmel Farms, Uddel, the Netherlands). Ferrets were screened for the presence of serum antibodies against Aleutian Disease virus, circulating seasonal influenza virus strains (A/H1N1, A/H3N2 and B) and the challenge virus (H5N1, A/Indonesia/5/2005), and only seronegative animals were used in the study. The animals were housed in study groups of 6 (prophylactic experiment) or 10 (therapeutic experiment).

Antibodies were administered by intravenous injection in the jugular vein. Viral challenge was performed intratracheally with 10^5^ TCID_50_ A/Indonesia/05/2005 in 3 mL of PBS [33]. Clinical observations were performed twice a day on days of intervention and once daily on other days. In the prophylactic experiment, animals were weighed 2 weeks before viral challenge (day −14), immediately prior to antibody administration (day −1), and after challenge (days 2, 4, and 5 or on the day of premature death). In the therapeutic experiment, animals were weighed on day −14 and day −2, immediately prior to challenge and antibody administration, and on days 2, 4 and the last study day. Body temperature was recorded every 15 minutes throughout both experiments using a device (DST micro-T, Star-Oddi, Reykjavik, Iceland) implanted in the peritoneal cavity 14 days before challenge.

The animal experiments were carried out in the central animal facilities of the Netherlands Vaccine Institute (NVI, Bilthoven) under conditions that meet the Dutch legal requirements for animal experimentation and are in accordance with the ‘Guide for the care and use of laboratory animals’, the recommendations of the Institute for Laboratory Animal Research (US National Institutes of Health), and Association for Assessment and Accreditation of Laboratory Animal Care International (AAALAC) standards.

### Viral Shedding

Viral titrations were performed as described elsewhere [34]. Briefly, pharyngeal and nasal swabs were collected from all animals at day −1, day 2, day 4, and day 5. Individual swabs were homogenized and resuspended in 3 ml medium and stored at −80°C until analysis. Viral titres were determined by virus titration on Madine Darby canine kidney (MDCK) cells. Viral shedding from the upper respiratory tract was analysed by calculating the proportion of ferrets with detectable levels of infectious virus in nasal and/or throat swabs relative to the number of living ferrets.

### Viral Load

After necropsy, the cranioventral, craniodorsal, caudoventral and caudodorsal sections of the right lung were collected from each animal, weighed, homogenized and resuspended in 3 ml medium, and stored at −80°C until analysis. Viral titres were determined after thawing of the tissue sections followed by homogenization and resuspension by virus titration on MDCK cells. In addition, viral titres in tissues of brain, spleen, liver, kidney, and plasma from animals that received either 30 mg/kg of CR6261 or CR3014 one day prior to challenge were determined using the same method.

### Pathology

A complete macroscopic post-mortem examination was performed on all animals. This included examination of the external surfaces and all orifices; the thoracic, abdominal and pelvic cavities with their associated organs and tissues; and the neck with its associated organs and tissues. Lungs were weighed and all lung lobes were inspected and lesions described. The left lung (including trachea) was collected during autopsy, inflated with 10% neutral buffered formalin for fixation/histology and microscopic examination. Paraffin embedded tissue sections (left cranial lobe, left caudal lobe, right cranial-, middle- and caudal lobes and accessory lobe) were stained with haematoxylin and eosin and assessed by light microscopy for aspects like congestion, emphysema, presence of foreign body, haemorrhagy, bronchioloalveolar hyperplasia and inflammation, and oedema.

### Anaesthesia

Prior to blood sampling, the taking of nose and throat swabs and euthanasia, the ferrets were anaesthetised with ketamin (25 mg/kg; i.m.). For implantation of temperature sensors, antibody administration and viral challenge, the animals were anaesthetized with a mixture of ketamin (12.5 mg/kg; i.m.) and domitor (7.5 µg/kg; i.m.), followed by antisedan (0.5 mg/kg; i.m.).

### Statistical Analyses

Survival times after viral challenge were analysed using the log-rank test and survival proportions using the Fisher's exact test. Body weight expressed as percentage change from baseline was calculated at the end of the study period (or earlier in the event of earlier death). Maximum body temperatures were observed during day one and these values were subsequently used for calculating the mean maximum body temperature for each group. Variables were analysed in an one-way analysis of variance (ANOVA) with post-hoc testing to compare to the control group using Dunnett's adjustment for multiple comparisons.

Lung weight and lung viral titres were compared across arms using ANOVA, with day of necropsy entered as a covariate. Differences between treatment groups were estimated using marginal means, with Sidak's adjustment for multiple comparisons. Statistical analyses were performed using SPSS version 15.0 (SPSS Inc. USA). Statistical significance level was set at α = 0.05.
